# Including oral diseases and conditions in universal health coverage

**DOI:** 10.2471/BLT.21.285530

**Published:** 2021-06-01

**Authors:** Stefan Listl, Carlos Quiñonez, Marko Vujicic

**Affiliations:** a Department of Dentistry, Quality and Safety of Oral Healthcare, Radboud University Medical Center, Nijmegen, Philips van Leydenlaan 25, 6525EX Nijmegen, Netherlands.; b Faculty of Dentistry, University of Toronto, Toronto, Canada.; c Health Policy Institute, American Dental Association, Chicago, United States of America.

The coronavirus disease 2019 (COVID-19) pandemic has increased the amount of government support required to meet people’s basic needs, including oral health and oral health care. Efforts towards universal health coverage (UHC) are only starting to include oral health and oral health care.^1^ Oral health and access to oral health care are fundamental elements of overall health and well-being, enabling individuals to successfully perform essential daily functions.^2^^,^^3^ Globally, oral diseases and conditions (dental caries, periodontal diseases, tooth loss and oral and pharyngeal cancers) represent some of the most preventable disease burdens; they also disproportionally affect poorer and marginalized groups.^2^ Untreated caries, severe periodontitis and tooth loss are among the 10 most prevalent conditions, globally affecting more than 3.5 billion people in 2017.^2^ The prevalence of dental caries is increasing in many low- and middle-income countries,^2^ while in some high-income countries, dental conditions account for 5.3% of loss in quality-adjusted life expectancy.^4^

Oral diseases have significant economic impact on individuals and societies in low- and middle-income countries as well as in high-income countries. Globally, dental diseases accounted for 356.80 billion United States dollars (US$) in direct costs and US$ 187.61 billion in indirect costs in 2015. These figures exclude costs associated with less severe states of dental diseases and oral and pharyngeal cancers.^5^ Oral diseases can hamper academic achievement in children and employment in adults, and may hinder workplace productivity.^2^^,^^5^ In some high-income countries, oral diseases lead to productivity losses comparable to those resulting from musculoskeletal disorders.^6^ Economic research indicates that the appearance of the mouth and teeth can influence hiring practices and affect employee earnings.^7^ In addition, oral diseases can worsen the burden of other diseases. For example, periodontal disease has been linked to poor glycaemic control among people living with diabetes and studies show that periodontal treatment can reduce total and diabetes-related health-care costs.^8^ Evidence suggests that in low- and middle-income countries, out-of-pocket expenditure for oral health care can lead to catastrophic health spending.^3^ Recent evidence also suggests limited affordability of oral health care in several high-income countries.^9^ Moreover, poor access to oral health care results in ineffective and inefficient use of primary and tertiary care.^10^

In their early stages, oral diseases and conditions are largely preventable and/or treatable with appropriate measures such as the use of fluorides; and reduction of sugar, tobacco and alcohol consumption. Such measures would lead to improvements in population oral health and overall well-being, generating substantial economic benefits through potentially reduced treatment costs and appropriate use of health-care resources, and fewer productivity losses in the labour market and beyond. The linkage between oral health and economic benefit has facilitated more focused discussions on the prioritization of some level of oral health-care coverage to all populations, particularly in the context of the COVID-19 pandemic.^11^^,^^12^ The negative consequences of inaction on oral health are twofold. First, the failure to attain a good balance between adequate investments in oral health care and investments for other health-care needs results in lower overall well-being than attainable with the available resources. Second, the failure to achieve best-possible use of available oral health-care resources results in compromised oral health and therefore reduced quality of life, reduced educational attainment, lower chances in the labour market, compromised social participation, exacerbation of other noncommunicable diseases and waste of resources due to avoidable treatment costs.

If oral health interventions are carefully chosen, the economic benefits of achieving UHC for oral health will outweigh the costs. Oral health programmes with high added value should be prioritized. To this end, the World Health Organization provides guidance to identify effective noncommunicable disease interventions and appropriate investment cases. 
